# Systematic Cys mutagenesis of FlgI, the flagellar P-ring component of *Escherichia coli*

**DOI:** 10.1099/mic.0.2007/013854-0

**Published:** 2008-03

**Authors:** Yohei Hizukuri, Seiji Kojima, Toshiharu Yakushi, Ikuro Kawagishi, Michio Homma

**Affiliations:** Division of Biological Science, Graduate School of Science, Nagoya University, Furo-Cho, Chikusa-Ku, Nagoya 464-8602, Japan

## Abstract

The bacterial flagellar motor is embedded in the cytoplasmic membrane, and penetrates the peptidoglycan layer and the outer membrane. A ring structure of the basal body called the P ring, which is located in the peptidoglycan layer, is thought to be required for smooth rotation and to function as a bushing. In this work, we characterized 32 cysteine-substituted *Escherichia coli* P-ring protein FlgI variants which were designed to substitute every 10th residue in the 346 aa mature form of FlgI. Immunoblot analysis against FlgI protein revealed that the cellular amounts of five FlgI variants were significantly decreased. Swarm assays showed that almost all of the variants had nearly wild-type function, but five variants significantly reduced the motility of the cells, and one of them in particular, FlgI G21C, completely disrupted FlgI function. The five residues that impaired motility of the cells were localized in the N terminus of FlgI. To demonstrate which residue(s) of FlgI is exposed to solvent on the surface of the protein, we examined cysteine modification by using the thiol-specific reagent methoxypolyethylene glycol 5000 maleimide, and classified the FlgI Cys variants into three groups: well-, moderately and less-labelled. Interestingly, the well- and moderately labelled residues of FlgI never overlapped with the residues known to be important for protein amount or motility. From these results and multiple alignments of amino acid sequences of various FlgI proteins, the highly conserved region in the N terminus, residues 1–120, of FlgI is speculated to play important roles in the stabilization of FlgI structure and the formation of the P ring by interacting with FlgI molecules and/or other flagellar components.

## INTRODUCTION

Bacteria swim by the rotation of flagella in a screw-like manner. The flagellar motor is embedded in the cytoplasmic membrane and the rotational power generated by the motor is transmitted to the helical flagellar filament through the hook. The flagellar motor, which is composed of numerous proteins, is divided into two parts, the rotor and the stator that surrounds the rotor. The rotor is mainly composed of the MS ring and the C ring, which is located on the cytoplasmic side of the MS ring. The stator is composed of the MotA/MotB complex, which contains at least four molecules of MotA and two molecules of MotB (Kojima & Blair, 2004), and functions as a proton channel (Blair & Berg, 1990; Stolz & Berg, 1991). About 10 of these stator units surround the rotor, and interactions between the stator and rotor are believed to generate the driving force for rotation (Reid *et al.*, 2006). The MotA/MotB stators assemble around the flagellar basal body, and the functional flagellar motor is established. It is thought that the peptidoglycan binding motif of MotB is involved in the assembly. A recent study has revealed that in *Escherichia coli*, the stator complexes incorporated into the motor are exchanged frequently with the one that floats on the cytoplasmic membrane near the motor (Leake *et al.*, 2006), suggesting that the interaction between the rotor and the stator is weak and/or temporary.

The P ring is one of the components of the basal body. In Gram-negative bacteria, it assembles around a proximal part of the basal body, and is thought to be attached to the peptidoglycan layer; it forms a stiff cylindrical structure to hold the central rod with the L ring, which assembles at the LPS (outer membrane) layer (Akiba *et al.*, 1991). The P ring is a part of the basal body, but is believed to be a non-rotating component to hold the rod as a bushing. The P ring is thought to consist of 26 copies of a single protein, FlgI (Jones *et al.*, 1990; Sosinsky *et al.*, 1992), which is expressed as a precursor form with a cleavable N-terminal 19 aa leader sequence, and exported to the periplasmic space via the Sec apparatus (Homma *et al.*, 1987; Jones *et al.*, 1989), where it assembles into the P ring surrounding the rod (Kubori *et al.*, 1992). The flagellar structure is constructed by a highly ordered process. First the MS ring is assembled at the cytoplasmic membrane as a base plate, then the C ring, the transport apparatus and the rod structure are assembled in turn. Next, the P- and L-ring structures are assembled around the rod, followed by the hook and the filament. Disruption of any flagellar component causes the assembly of flagellar structure to arrest; a disruption in FlgI causes a motility defect because the flagellar construction terminates at the rod structure. Recently, we revealed that the intramolecular disulfide bond formation in FlgI is not necessary for P-ring assembly but is important to protect against degradation of the protein (Hizukuri *et al.*, 2006).

**Fig. 1. f1:**
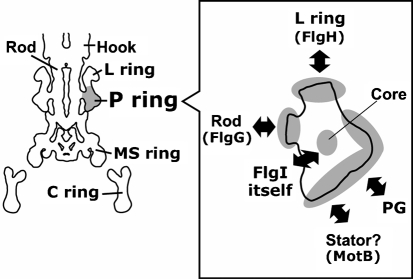
Information about the P-ring protein FlgI; possible interactions between the P-ring protein FlgI and other components. In the left-hand image, cylindrically averaged flagellar structure reported by DeRosier (1998) is outlined. The estimated P-ring region is shaded. The drawing of the FlgI monomer displayed in the right-hand box is an estimated shape. PG, peptidoglycan.

Various interactions have been speculated for the P-ring protein FlgI in the flagellar basal body (Fig. 1). To expand our knowledge of the P-ring structure and to understand the spatial arrangement around the rod in the periplasmic space, we constructed and characterized a series of systematically Cys-substituted *E. coli* FlgI variants. Among 32 FlgI Cys variants constructed, the protein amounts of five FlgI variants were significantly decreased, and cells carrying five of the variants showed reduced motility. We further characterized the variants using a thiol-specific reagent to investigate which residue of the protein was exposed to solvent on the protein surface. Interestingly, this work showed that the residues of FlgI that can be labelled never overlap with the residues found to be important for protein stability or motility.

## METHODS

### Bacterial strains, growth conditions, and media.

The *E. coli* strains used in this work are listed in Table 1. To delete the *cat* gene cassette of the Δ*flgI* : : *cat* strain YZ1 (Hizukuri *et al.*, 2006), we used the method described by Datsenko & Wanner (2000), and the constructed strain was named YZ11. The Δ*motAB* : : *cat* strain YS5 was kindly provided by Yoshiyuki Sowa (Oxford University). To construct a Δ*flgI* Δ*motAB* : : *cat* triple deletion strain, the Δ*motAB* : : *cat* region of YS5 was transferred into strain YZ11 by using P1 phage (Silhavy *et al.*, 1984), and the resultant strain was named YZ12-1. *E. coli* cells were cultured at 37 °C in LB medium (1 % Bacto tryptone, 0.5 % yeast extract, 0.5 % NaCl) or at 30 °C in TG medium (1 % Bacto tryptone, 0.5 % NaCl, 0.5 %, w/v, glycerol). When necessary, ampicillin and kanamycin were added to a final concentration of 50 μg ml^−1^.

### Construction of plasmids.

Routine DNA manipulations were carried out according to standard procedures (Sambrook *et al.*, 1989). The plasmids used in this work are listed in Table 1. To construct the pYZ301 plasmid, a 1.4 kb *Kpn*I–*Sph*I fragment containing the *E. coli flgI* gene was cut out of pYZ201 (Hizukuri *et al.*, 2006) and inserted into the corresponding sites of the vector pSU38. To obtain a series of plasmids expressing FlgI Cys variants, we performed site-directed mutagenesis on pYZ301 by which a full length of plasmid DNA was amplified by *PfuUltra* High-Fidelity DNA Polymerase (Stratagene) using a pair of complementary primers carrying a mutagenized codon, and was then digested by *Dpn*I. To construct the pJN726 plasmid, a 2.4 kb *Sal*I–*Sal*I fragment containing the *motAB* genes was cut out of pYA6022 (Asai *et al.*, 2003) and inserted into the vector pBAD24.

### Motility assays.

Swarming motility was assayed as follows. Overnight culture (2 μl) (grown on LB medium at 37 °C) was dropped on a soft agar T broth plate (1 % Bacto tryptone, 0.5 % NaCl, 0.27 % Bacto agar) containing 50 μg ml^−1^ each of ampicillin and kanamycin and 0.04 % l-arabinose. If necessary, 5 mM DTT was added to agar plates. The plates were incubated at 30 °C for the time indicated for each experiment. Relative swarm size of the FlgI Cys mutant was calculated by normalizing to the diameter of the swarm ring of the wild-type FlgI-expressing cells after subtracting the diameter of the swarm ring of the vector-containing cells.

### Detection of FlgI.

Immunoblot analysis using anti-*E. coli* FlgI antibodies (FlgI346) was performed as described previously (Hizukuri *et al.*, 2006).

### Cysteine modification by methoxypolyethylene glycol 5000 maleimide (mPEG-maleimide).

An overnight culture (grown on LB medium at 37 °C) was inoculated at a 50-fold dilution into TG medium containing 50 μg ml^−1^ ampicillin and kanamycin and 0.04 % l-arabinose, and cultured at 30 °C. At the exponential growth phase, 300 μl of cultured cells (OD_660_=1.0) was harvested by centrifugation (9500 ***g***, 5 min). The cells were suspended in 1 ml Wash Buffer (10 mM potassium phosphate buffer, pH 7.0, containing 0.1 mM EDTA-K), centrifuged again and resuspended in 25 μl MLM Buffer (Wash Buffer containing 10 mM dl-lactate/KOH and 0.1 mM l-methionine). The thiol-specific reagent mPEG-maleimide (Fluka) was suspended in DMSO as a 40 mM stock solution and stored at −20 °C in the dark. Twenty-five microlitres of mPEG-maleimide reaction buffer (MLM Buffer containing 4 mM mPEG-maleimide, prepared freshly before each experiment) was added to cell suspensions and mixed well, then incubated with shaking at 37 °C for 30 min. To terminate the reaction, 5 μl *β*-mercaptoethanol was added and then 5 μl 10 % SDS. The sample solution was boiled at 100 °C for 5 min and mixed with 15 μl 5× SDS loading buffer containing *β*-mercaptoethanol. An aliquot of 5 μl was used for SDS-PAGE.

## RESULTS

### Protein amounts and cross-linked products of FlgI Cys variants

We constructed the *E. coli* Δ*flgI* Δ*motAB* : : *cat* triple deletion strain YZ12-1. The constructed strain showed no motility in either liquid or soft agar medium, and no flagella were observed by electron microscopy (data not shown). When transformed with two plasmids, one harbouring the *flgI* gene and the other the *motAB* genes, YZ12-1 cell motility was almost the same as that of wild-type cells in liquid medium (data not shown). Hereafter, we refer to this complemented strain as the wild-type FlgI-expressing strain. In the future we plan to investigate the interaction between the P ring and the MotA/MotB stator complex; however, we focused our current investigation on the P-ring structure and its function.

**Fig. 2. f2:**
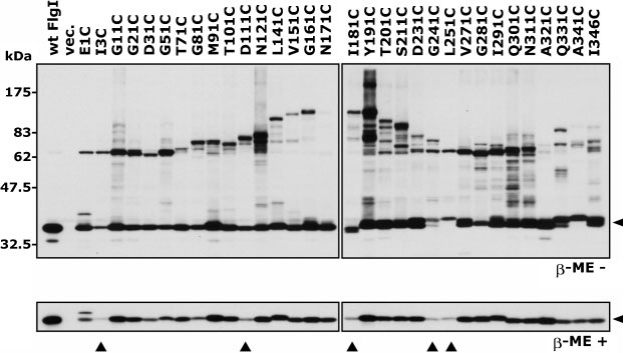
Detection of products of systematically substituted FlgI Cys variants. The FlgI proteins from YZ12-1 (Δ*flgI* Δ*motAB* : : *cat*) cells harbouring pJN726 (MotAB) and pYZ301 (wt FlgI), pSU38 (vec.) or the pYZ301 derivatives containing a series of FlgI Cys variants were detected by immunoblotting using anti-FlgI antibodies. Upper panel, without *β*-mercaptoethanol (*β*-ME -); lower panel, with *β*-mercaptoethanol (*β*-ME +). The arrowheads on the right-hand side indicate the monomer of the mature form of FlgI (36 kDa). Filled triangles below the lower panel indicate the FlgI Cys variants for which the amount of protein was significantly lower than that of the wild-type.

We systematically constructed a series of FlgI Cys variants. We designed cysteine substitutions every 10th residue in the mature form of FlgI (the numbers correspond to the positions of the amino acid residues in the mature form of FlgI). Two additional mutants were also designed in which Ile^3^ and Ile^346^ were substituted with cysteine (Fig. 6). FlgI protein has two native cysteine residues, at positions 254 and 338, and they remained intact in our Cys-substituted variants. From the 37 candidates designed, we obtained 32 FlgI Cys variants, but were unable to generate D41C, Q61C, A131C, Q221C or L261C. We examined the protein amounts of the FlgI Cys variants by immunoblot analysis using anti-FlgI antibodies (Fig. 2). Most of the FlgI variants showed slightly decreased amounts of product compared to wild-type FlgI. However, the protein amounts of five variants, I3C, D111C, I181C, G241C and L251C, were significantly decreased compared to the other variants (Fig. 2, *β*-ME +, filled triangles). We have reported that FlgI C254A, FlgI C338A and the double mutant seem to be more susceptible to degradation (Hizukuri *et al.*, 2006), so we speculate that FlgI Gly^241^ and Leu^251^ (which is located near Cys^254^) affect the susceptibility of the protein to degradation when replaced with Cys. In FlgI E1C, an additional band that was larger (by ∼2 kDa) than the estimated monomer band was detected. The larger protein may be a precursor form (38 kDa) of the mature form of FlgI (36 kDa) because replacement of the residue next to a signal cleavage site probably affects the cleavage efficiency. In the absence of the reductant *β*-mercaptoethanol, disulfide cross-linked products were detected in most of the FlgI Cys variants (Fig. 2, *β*-ME -). The apparent molecular masses of the cross-linked products showed a wave-like pattern with a peak at residues ∼160–190. The variants with Cys replacements at the N- or C-terminal regions had the approximate estimated size of dimers (72 kDa). On the other hand, replacements near the central region caused decreased mobility of the bands, probably because the cross-linked dimers at the middle positions formed aberrant shapes. It is worth noting that FlgI Y191C showed a large number of cross-linked products, whereas FlgI N171C and FlgI A321C showed almost no cross-linked products.

### Effects of the FlgI Cys variants on motility

**Fig. 3. f3:**
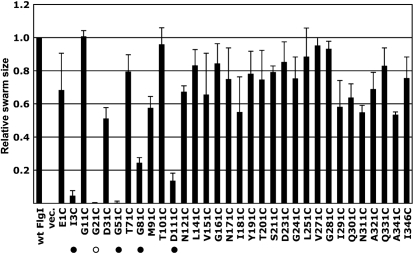
Motility of the FlgI Cys variant-expressing cells. The relative swarm size is shown of YZ12-1 (Δ*flgI* Δ*motAB* : : *cat*) cells harbouring pJN726 (MotAB) and pYZ301 (wt FlgI), pSU38 (vec.) or pYZ301 derivatives containing a series of FlgI Cys variants. A drop (2 μl) of overnight culture was inoculated on 0.27 % soft agar T broth plates, which were incubated at 30 °C for an appropriate period of time. The swarm assays were repeated three times independently and the relative swarm sizes calculated in each experiment were averaged. •, Weakly motile mutant; ○, non-motile mutant.

**Fig. 4. f4:**
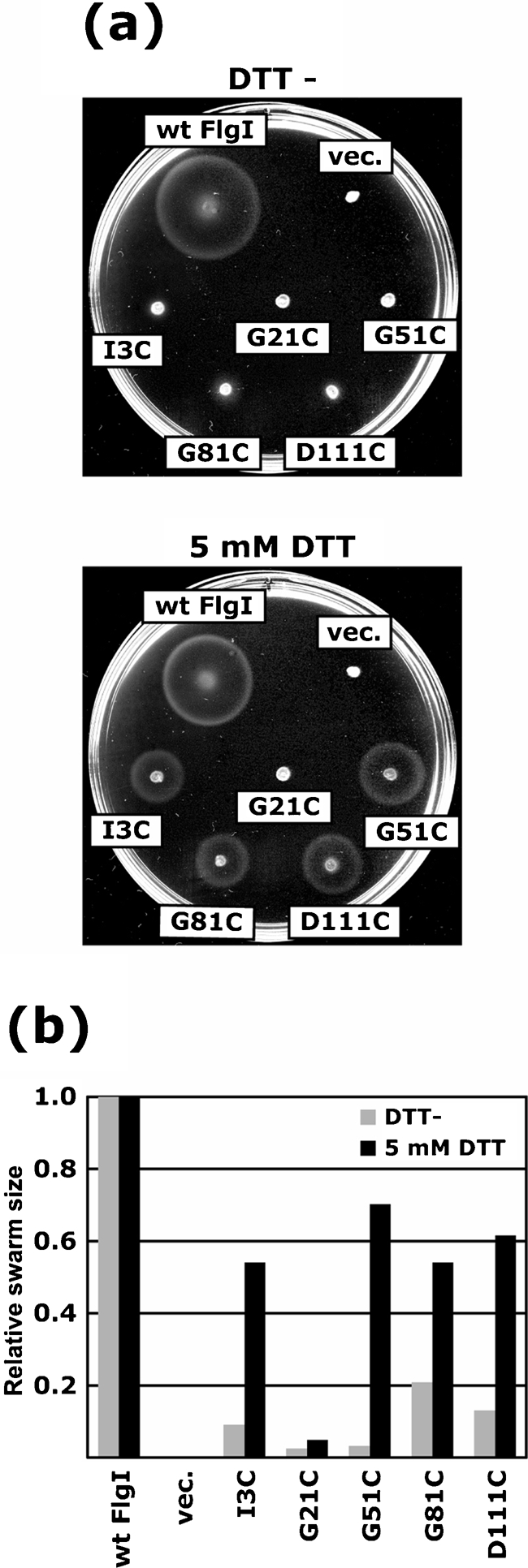
Effect of DTT on motility of the FlgI Cys mutants. (a) Swarms of YZ12-1 (Δ*flgI* Δ*motAB* : : *cat*) cells harbouring pJN726 (MotAB) and pYZ301 (wt FlgI), pSU38 (vec.) or pYZ301 derivatives containing the FlgI Cys variants I3C, G21C, G51C, G81C and D111C. A drop (2 μl) of overnight culture was inoculated on 0.27 % soft agar T broth plates without (upper panel) or with (lower panel) 5 mM DTT and plates were incubated at 30 °C for 8 h. (b) Relative swarm sizes from (a). Grey bars, without DTT; black bars, with 5 mM DTT. The swarm size of cells producing wild-type FlgI was used for normalization of the mutants under the equivalent conditions.

Defects in FlgI cause a failure of P-ring assembly and result in the termination of flagellar formation after rod construction. To assess the effects of the Cys replacements in FlgI on P-ring assembly, we first examined swarming ability in soft agar plates. We measured swarm ring sizes after sufficient incubation and calculated the relative swarm rates for each FlgI Cys mutant against that of wild-type FlgI (Fig. 3). Most of the FlgI Cys mutants retained swarming ability, but five mutants, FlgI I3C, G21C, G51C, G81C and D111C, had significantly decreased swarm rates (see also Fig. 4a[Fig f4], upper panel). When we observed swimming ability in liquid medium using dark-field microscopy, cells carrying each of these five mutations except G21C were motile, although the fractions of motile cells were extremely low (<5 %); cells carrying the G21C mutation completely lost swimming ability. When we observed the cells by electron microscopy, the mutant cells expressing FlgI G21C were shown to be completely non-flagellate (data not shown), implying that the Gly^21^ residue of FlgI is critical for P-ring assembly.

We investigated the effects of reducing agents such as DTT on the motility of the weakly motile mutants FlgI I3C, G51C, G81C and D11C, and the non-motile mutant G21C (Fig. 4). In the absence of DTT, these five mutants showed poor or no motility, as described above. On the other hand, when 5 mM DTT was added to the motility agar, the swarm ring size of the wild-type cells was slightly decreased, but the four weakly motile mutants had significantly restored swarming abilities. The non-motile mutant G21C remained completely non-motile. These results suggest that the motility defects of the four weakly motile mutants were caused by the formation of an incorrect disulfide bond by the replacement Cys either within FlgI itself or with another Cys-containing protein(s).

### Thiol modification of the FlgI Cys variants by mPEG-maleimide

**Fig. 5. f5:**
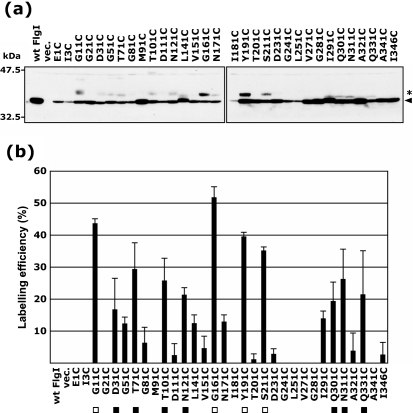
Cysteine modification of the FlgI Cys variants by mPEG-maleimide. (a) mPEG-maleimide-labelled FlgI proteins derived from YZ12-1 (Δ*flgI* Δ*motAB* : : *cat*) cells harbouring pJN726 (MotAB) and pYZ301 (wt FlgI), pSU38 (vec.) or pYZ301 derivatives containing the FlgI Cys variants were detected by immunoblotting using anti-FlgI antibodies. For the mPEG-maleimide modification, cultures in the exponential growth phase were harvested, washed and incubated with 2 mM mPEG-maleimide at 37 °C for 30 min. After terminating the reaction, the samples were mixed with SDS loading buffer containing *β*-mercaptoethanol. The arrowhead on the right-hand side indicates the mature form of the FlgI monomer (36 kDa) and the asterisk indicates mPEG-maleimide-labelled FlgI, as judged by the ∼5 kDa shift of the monomer band. (b) mPEG-maleimide labelling efficiency was obtained by dividing the intensity of the shifted band by the total band intensity of FlgI. ▪, Well-labelled (>30 % labelling efficiency); □, moderately labelled (>15 % labelling efficiency).

To obtain structural information about FlgI, we examined the cysteine modification of the FlgI Cys variants using mPEG-maleimide, which is a membrane-impermeable thiol-specific reagent that carries an attached polyethylene glycol and has a high molecular mass of ∼5000 Da (Akiyama *et al.*, 2004). We detected the reaction by mobility shifts of the bands on SDS-PAGE gels (Fig. 5a[Fig f5]). The band of wild-type FlgI did not shift in mobility, suggesting that the two native Cys residues of FlgI were not accessed by mPEG-maleimide, probably because they formed an intramolecular disulfide bond. On the other hand, some of the FlgI Cys variants showed ∼5 kDa shift of the monomer band (36 kDa), indicating that the additional Cys residue was accessible to mPEG-maleimide, which means that the additional Cys residues are likely to be exposed to solvent on the surface of the protein. To analyse accessibility further, we quantified the labelling efficiencies of each FlgI Cys variant, which are given relative to the total amount of FlgI present (Fig. 5b[Fig f5]). Based on this analysis, we could classify these variants into three groups: the high labelling efficiency group (>30 %, open rectangles), FlgI G11C, G161C, Y191C and S211C; the moderate labelling efficiency group (>15 %, closed rectangles), FlgI D31C, T71C, T101C, N121C, Q301C, N311C and Q331C; and the low labelling efficiency and non-labelling group (<15 %), which contains the other mutants.

### Alignment of the amino acid sequences of the P-ring protein FlgI from various species

The replaced residues that impaired cell motility in this work were localized around the N terminus of FlgI (Ile^3^, Gly^21^, Gly^51^, Gly^81^ and Asp^111^; Fig. 3). To assess the importance of the N terminus of FlgI, we aligned the amino acid sequences of FlgI obtained from various flagellated bacteria (Supplementary Fig. S1). Multiple alignments revealed that the amino acid sequences of FlgI were well conserved among various bacteria, except the N-terminal leader sequence, and in particular, the sequences of the N-terminal region of FlgI (Arg^2^–Asp^31^ and Lys^63^–Gly^120^) were extremely well conserved. Replacement of Gly^21^ with Cys, which is in the most highly conserved region, caused the most severe phenotype, non-motility. The five residues whose mutation impaired cell motility were all positioned around this N-terminal highly conserved region. From this alignment analysis and our experimental results, the N-terminal highly conserved region of FlgI is suggested to play important roles in various functions, such as maintenance of the structure of FlgI and formation of an interface with other flagellar proteins or with itself.

## DISCUSSION

**Fig. 6. f6:**
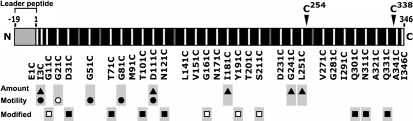
Profiles of the FlgI Cys mutants. The grey bar at the N terminus indicates the leader peptide, which is cleaved when the protein is exported to the periplasm. The white vertical bands indicate the residues substituted with Cys in this study, while the grey vertical bands indicate the substitutions that we were unable to generate. C^254^ and C^338^ are shown above to indicate the positions of the native cysteine residues in FlgI. Profiles are shown under the name of each variant: Amount, the residues for which the protein amount was decreased when substituted with Cys; Motility, the residues that caused decreased (•) or completely disrupted (○) motility of the cells; Modified, the residues that were well- (□) or moderately (▪) labelled by mPEG-maleimide. The residues well- or moderately labelled by mPEG-maleimide (rectangles) never overlap with the residues that affect their protein amount (triangles) or the motility of cells (circles).

In this study, we characterized a series of FlgI Cys-substituted mutants with respect to protein amount, motility of the cells, and modification by a thiol-specific reagent. The results of this work are summarized in Fig. 6. Among the mutations, the well- or moderately labelled residues (rectangles) never overlapped with the residues that affected their protein amount (triangles) or the motility of cells (circles). This may suggest that the important residues for protein folding or P-ring assembly are not exposed to solvent on the surface of the protein. This seems to be reasonable, because residues important for protein folding or assembly are likely to be located inside the protein (i.e. forming the core) or at the protein–protein interface. FlgI is secreted to the periplasmic space and then assembled around the rod of the flagellar basal body to form the P-ring structure. FlgI interacts with other FlgI molecules to form the P ring, with the L-ring protein that is located above the P ring, with rod proteins, with peptidoglycan (PG), and possibly with the MotA/B stator complex or other components in the periplasmic space. Speculative interactions of FlgI are illustrated in Fig. 1. FlgI is predicted to form strong interactions with FlgI itself or FlgH, the L-ring protein. In *Salmonella enterica* serovar Typhimurium, the P and L rings form an extremely stiff cylindrical structure. Only the L–P ring complex remains after 7.5 M urea treatment, during which all of the other flagellar components dissociate (Akiba *et al.*, 1991). Based on this evidence, it has been inferred that the interactions of FlgI–FlgI and FlgI–FlgH are probably very strong.

FlgI may also interact with FlgG, a distal rod protein. In the process of P-ring assembly, secreted FlgI is predicted to recognize the rod structure in the periplasm and associate with the rod. The rod is a part of the rotor structure, but the P ring is believed to be a part of the stator that supports the rod as a bushing and allows the rotor to run smoothly. Therefore, it has been predicted that the FlgI–FlgG interaction is temporary and/or very weak. The P ring is located in the peptidoglycan layer (and so is named the P ring), and it may interact with the peptidoglycan layer. Considering the role of the P ring, it is very likely that the P ring is fixed in the peptidoglycan layer to stabilize rotation of the motor.

The highly conserved region in the N terminus, residues 1–120, of FlgI is suggested to play an important role, such as in stabilizing the structure of FlgI or forming an interface with other FlgI proteins or other flagellar components, i.e. FlgH. The role of the conserved region is not known, but it may be important to maintain FlgI structure, because there are many Gly and Pro residues in this region. The G161C variant was the most accessible to the cysteine modification reagent. When the hook basal bodies (HBBs) isolated from cells expressing FlgI G161C were treated with mPEG-maleimide, FlgI was labelled and showed band shifting (data not shown). In addition, FlgI Y191C showed numerous cross-linked products compared to other variants (Fig. 2). These results may suggest that the central region of FlgI is exposed to the outer surface of the P ring. In our previous work, we reported that the replacement of the native Cys residues of FlgI (Cys^254^ and/or Cys^338^) with Ala has little effect on motility but results in a significantly decreased amount of protein (Hizukuri *et al.*, 2006). We concluded that the intramolecular disulfide bond formed between Cys^254^ and Cys^338^ is required to prevent the degradation of protein. Here, we showed that the amount of FlgI protein is decreased for FlgI G241C or L251C, but is not changed in FlgI Q331C or A341C. The FlgI C254A mutation has a more severe effect on both flagellar motility and protein amount than the FlgI C338A mutation (Hizukuri *et al.*, 2006). These results may suggest that the amino acids around Cys^254^ are more important for protein folding or protection against degradation than those around Cys^338^.

Recently, a novel structure, named the T ring, was discovered in the flagellar basal body of *Vibrio alginolyticus* (Terashima *et al.*, 2006). The T ring is located on the periplasmic side of the P ring and is composed of MotX and MotY, which are essential proteins for motor function. The T ring is proposed to interact with the PomA/PomB stator complex, which is homologous to the MotA/MotB complex, by the interaction between MotX and PomB. The T ring has an important role in the incorporation and stabilization of the stator (Okabe *et al.*, 2005; Terashima *et al.*, 2006). *E. coli* and *Salmonella* species do not have a T ring or protein homologues of MotX or MotY. We think that the P ring of *E. coli* might have a similar role to that of the T ring for incorporation or stabilization of the MotA/MotB stator in the motor. The C-terminal peptidoglycan binding (PGB) motif of MotB is believed to anchor to the peptidoglycan layer via the central flexible linker region of MotB and to stabilize the stator complex during rotation. The P ring is also located in the peptidoglycan layer; thus, the stator complex may be associated with the P ring via the PGB motif of MotB when it assembles and functions around the motor. In future studies, we will assess the possible interactions between the P ring and MotB.

## Figures and Tables

**Table 1. t1:** Strains and plasmids

**Strain or plasmid**	Genotype or description*	**Reference or source**
**Strains**		
RP437	F^−^*thi thr leu his met eda rpsL* wild-type for chemotaxis	Parkinson & Houts (1982)
YZ1	RP437 Δ*flgI* : : *cat*	Hizukuri *et al.* (2006)
YZ11	RP437 Δ*flgI*	This work
YS5	RP437 Δ*motAB* : : *cat*	Yoshiyuki Sowa
YZ12-1	RP437 Δ*flgI* Δ*motAB* : : *cat*	This work
**Plasmids**		
pBAD24	P_BAD_*araC* Amp^r^	Guzman *et al.* (1995)
pSU38	P_lac_*lacZα* Km^r^	Bartolome *et al.* (1991)
pYZ201	pBAD24 *flgI*	Hizukuri *et al.* (2006)
pYZ301	pSU38 *flgI*	This work
pYA6022	pSU41 *motAB*	Asai *et al.* (2003)
pJN726	pBAD24 *motAB*	This work

*Abbreviations: Amp^r^, ampicillin resistant; Km^r^, kanamycin resistant; P_BAD_, *araBAD* promoter; P_lac_, *lac* promoter.

## Abbreviations

- mPEG-maleimide, methoxypolyethylene glycol 5000 maleimide

## References

[r1] Akiba, T., Yoshimura, H. & Namba, K. (1991). Monolayer crystallization of flagellar L-P rings by sequential addition and depletion of lipid. Science 252, 1544–1546. 2047860 10.1126/science.2047860

[r2] Akiyama, Y., Kanehara, K. & Ito, K. (2004). RseP (YaeL), an *Escherichia coli* RIP protease, cleaves transmembrane sequences. EMBO J 23, 4434–4442. 15496982 10.1038/sj.emboj.7600449 PMC526465

[r3] Asai, Y., Yakushi, T., Kawagishi, I. & Homma, M. (2003). Ion-coupling determinants of Na^+^-driven and H^+^-driven flagellar motors. J Mol Biol 327, 453–463. 12628250 10.1016/s0022-2836(03)00096-2

[r4] Bartolome, B., Jubete, Y., Martinez, E. & de la Cruz, F. (1991). Construction and properties of a family of pACYC184-derived cloning vectors compatible with pBR322 and its derivatives. Gene 102, 75–78. 1840539 10.1016/0378-1119(91)90541-i

[r5] Blair, D. F. & Berg, H. C. (1990). The MotA protein of *E. coli* is a proton-conducting component of the flagellar motor. Cell 60, 439–449. 2154333 10.1016/0092-8674(90)90595-6

[r6] Datsenko, K. A. & Wanner, B. L. (2000). One-step inactivation of chromosomal genes in *Escherichia coli* K-12 using PCR products. Proc Natl Acad Sci U S A 97, 6640–6645. 10829079 10.1073/pnas.120163297 PMC18686

[r7] DeRosier, D. J. (1998). The turn of the screw: the bacterial flagellar motor. Cell 93, 17–20. 9546387 10.1016/s0092-8674(00)81141-1

[r8] Guzman, L. M., Belin, D., Carson, M. J. & Beckwith, J. (1995). Tight regulation, modulation, and high-level expression by vectors containing the arabinose P_BAD_ promoter. J Bacteriol 177, 4121–4130. 7608087 10.1128/jb.177.14.4121-4130.1995 PMC177145

[r9] Hizukuri, Y., Yakushi, T., Kawagishi, I. & Homma, M. (2006). Role of the intramolecular disulfide bond in FlgI, the flagellar P-ring component of *Escherichia coli*. J Bacteriol 188, 4190–4197. 16740925 10.1128/JB.01896-05 PMC1482947

[r10] Homma, M., Komeda, Y., Iino, T. & Macnab, R. M. (1987). The *flaFIX* gene product of *Salmonella typhimurium* is a flagellar basal body component with a signal peptide for export. J Bacteriol 169, 1493–1498. 3549691 10.1128/jb.169.4.1493-1498.1987 PMC211974

[r11] Jones, C. J., Homma, M. & Macnab, R. M. (1989). L-, P-, and M-ring proteins of the flagellar basal body of *Salmonella typhimurium*: gene sequences and deduced protein sequences. J Bacteriol 171, 3890–3900. 2544561 10.1128/jb.171.7.3890-3900.1989 PMC210140

[r12] Jones, C. J., Macnab, R. M., Okino, H. & Aizawa, S. (1990). Stoichiometric analysis of the flagellar hook-(basal-body) complex of *Salmonella typhimurium*. J Mol Biol 212, 377–387. 2181149 10.1016/0022-2836(90)90132-6

[r13] Kojima, S. & Blair, D. F. (2004). Solubilization and purification of the MotA/MotB complex of *Escherichia coli*. Biochemistry 43, 26–34. 14705928 10.1021/bi035405l

[r14] Kubori, T., Shimamoto, N., Yamaguchi, S., Namba, K. & Aizawa, S. (1992). Morphological pathway of flagellar assembly in *Salmonella typhimurium*. J Mol Biol 226, 433–446. 1640458 10.1016/0022-2836(92)90958-m

[r15] Leake, M. C., Chandler, J. H., Wadhams, G. H., Bai, F., Berry, R. M. & Armitage, J. P. (2006). Stoichiometry and turnover in single, functioning membrane protein complexes. Nature 443, 355–358. 16971952 10.1038/nature05135

[r16] Okabe, M., Yakushi, T. & Homma, M. (2005). Interactions of MotX with MotY and with the PomA/PomB sodium ion channel complex of the *Vibrio alginolyticus* polar flagellum. J Biol Chem 280, 25659–25664. 15866878 10.1074/jbc.M500263200

[r17] Parkinson, J. S. & Houts, S. E. (1982). Isolation and behavior of *Escherichia coli* deletion mutants lacking chemotaxis functions. J Bacteriol 151, 106–113. 7045071 10.1128/jb.151.1.106-113.1982 PMC220204

[r18] Reid, S. W., Leake, M. C., Chandler, J. H., Lo, C. J., Armitage, J. P. & Berry, R. M. (2006). The maximum number of torque-generating units in the flagellar motor of *Escherichia coli* is at least 11. Proc Natl Acad Sci U S A 103, 8066–8071. 16698936 10.1073/pnas.0509932103 PMC1472430

[r19] Sambrook, J., Fritsch, E. F. & Maniatis, T. (1989). *Molecular Cloning: a Laboratory Manual*, 2nd edn. Cold Spring Harbor, NY: Cold Spring Harbor Laboratory.

[r20] Silhavy, T. J., Berman, M. L. & Enquist, L. W. (1984). *Experiments with Gene Fusions*. Cold Spring Harbor, NY: Cold Spring Harbor Laboratory.

[r21] Sosinsky, G. E., Francis, N. R., DeRosier, D. J., Wall, J. S., Simon, M. N. & Hainfeld, J. (1992). Mass determination and estimation of subunit stoichiometry of the bacterial hook–basal body flagellar complex of *Salmonella typhimurium* by scanning transmission electron microscopy. Proc Natl Acad Sci U S A 89, 4801–4805. 1594581 10.1073/pnas.89.11.4801 PMC49175

[r22] Stolz, B. & Berg, H. C. (1991). Evidence for interactions between MotA and MotB, torque-generating elements of the flagellar motor of *Escherichia coli*. J Bacteriol 173, 7033–7037. 1938906 10.1128/jb.173.21.7033-7037.1991 PMC209062

[r23] Terashima, H., Fukuoka, H., Yakushi, T., Kojima, S. & Homma, M. (2006). The *Vibrio* motor proteins, MotX and MotY, are associated with the basal body of Na^+^-driven flagella and required for stator formation. Mol Microbiol 62, 1170–1180. 17038120 10.1111/j.1365-2958.2006.05435.x

